# Increasing Fruit and Vegetable Intake of Primary School Children in a Quasi-Randomized Trial: Evaluation of the Three-Year School-Based Multicomponent Intervention

**DOI:** 10.3390/nu14194197

**Published:** 2022-10-08

**Authors:** Ana Ilić, Ivana Rumbak, Ružica Brečić, Irena Colić Barić, Martina Bituh

**Affiliations:** 1 Department of Food Quality Control, Faculty of Food Technology and Biotechnology, University of Zagreb, Pierottijeva 6, 10000 Zagreb, Croatia; 2 Department of Marketing, Faculty of Economics and Business, University of Zagreb, J.F. Kennedy 6, 10000 Zagreb, Croatia

**Keywords:** child, eating behavior, fruit intake, nutrition intervention, school setting, vegetable intake, public health

## Abstract

Insufficient consumption of fruit and vegetables was found in primary school children. To address this problem, a three-year school-based multicomponent intervention was conducted in 14 primary schools in the City of Zagreb. The aim of the study was therefore to evaluate one of the primary goals of the intervention—the increase in fruit and vegetable intake among primary school children. A total of 681 children were allocated to the intervention (n = 300 in the control group and n = 381 in the intervention group). The intervention included 23 interactive classroom workshops, 10 cross-curricular activities, 13 homework challenges, visual exposure with educational posters in classrooms, parent education via the website, and the implementation of new dishes into the school food system. Fruit and vegetable intake was assessed using a semi-quantitative food frequency questionnaire at baseline and after the intervention. Data were analyzed by per-protocol analysis. The study involved 259 children (50.2% girls; age 7.7 ± 0.4 years; n = 116 in the control group and n = 143 in the intervention group) who completed a food intake frequency questionnaire at both time points. Children in the intervention group showed a significant increase (*p* < 0.001) in total daily fruit and vegetable intake (before: 332.1 ± 164.9 g; after: 430.1 ± 186.7 g) compared to the control group (before: 350.2 ± 187.5; after: 382.6 ± 196.8) after the intervention. The increase in fruit and vegetable intake was achieved in 89% of children, while 25% more children reached the daily recommendation of 400 g. The use of the multicomponent intervention showed potential to increase fruit and vegetable intake in primary school children.

## 1. Introduction

The health benefits of consuming fruit and vegetables are well known, both because of their nutrient composition and because of the synergistic effects of biologically active compounds [1,2,3]. Moreover, a diet rich in plant foods is considered a sustainable dietary pattern that has a positive impact on the environment [4]. Nevertheless, insufficient consumption of fruit and vegetables is observed worldwide, especially among children [5,6,7,8]. In particular, in Europe, it is estimated that 27% to 75% of children (6–9 years) consume fruit and 18% to 70% of children consume vegetables daily [5].

In response to this problem, various educational strategies have been developed aimed at increasing the consumption of fruit and vegetables in children. It is quite difficult to compare the effects of the different interventions on eating behavior because they have been implemented in primary school children with different activities. However, they are all considered to have had a moderate effect on increasing fruit consumption and a small effect on increasing vegetable consumption [9,10]. According to the available literature, multicomponent nutrition interventions have a greater effect than single-component interventions. In this context, multicomponent interventions include changes in the children’s environment, targeted education, and other activities such as cooking classes, homework, parent education, reinforcement, etc. [9,10,11,12,13,14,15]. Apart from the components included in the intervention, it is necessary to choose the appropriate theoretical framework for eating behavior change. Theory-based interventions have a greater impact on changing eating behavior in children compared to non-theory-based interventions [14,15,16]. Regardless of the type of intervention, it is necessary to know the needs of the community in which the intervention is implemented. In addition, knowledge of the determinants of the eating behavior of the population could influence the success of the implementation of the intervention. Certainly, the intervention should be placed within the social and cultural framework of the community in which it is implemented [11,16,17]. Common criticisms of the evaluation of the interventions in the available reviews and meta-analyses are that they were carried out on a small number of subjects, that the interventions were of short duration, that the period for monitoring behavioral changes after the end of the intervention was too short, and that there was a difference in the season in which the data on eating habits were collected before and after the intervention [9,10].

The WHO European Childhood Obesity Surveillance Initiative (COSI) found that only 33.8% of 8-year-old children in Croatia consumed fruit daily and only 16.7% consumed vegetables daily [18], which was the first indicator of the need to design and implement a nutrition intervention for primary school children. In addition, it was found that primary school children in Zagreb wasted more than a quarter of the served dishes with vegetables from school meals. The main reason why children wasted the served food was that they did not like the taste of the food [19]. Accordingly, the first nutrition intervention, “Nutri-školica,” was designed and implemented for three years in schools in Zagreb, Croatia, with the aim of increasing the consumption and preferences of fruits and vegetables among primary school children (7–9 years old). The intervention was planned in accordance with the knowledge about the advantages and disadvantages of existing interventions and adapted to the socio-cultural environment of the children. Children in the lower grades of primary school in Croatia can spend up to 8 h in school [20]. Therefore, the intervention was implemented in school to reduce the burden of children and increase the influence of peers and school staff on the development of children’s eating habits [21]. In addition, children can eat one to three school meals depending on the length of the lessons [22], but it was found that school menus did not contain enough fruit and vegetables [23]. Therefore, the educational component of the intervention was accompanied by changes in school menus. Recently, it was found that children who ate school meals and children who did not eat school meals obtained more than 35% of their daily energy intake from minimally processed foods, as well as ultra-processed foods [24], indicating the need for parental education. Parental involvement in school-based intervention (e.g., education, cooking activities, homework, etc.) showed greater reach in changing eating behaviors [11]. Although fast food is ubiquitous in children’s diets, most meals in Croatia still consist of cooked dishes [25], which facilitates the implementation of intervention components related to culinary and taste challenges with new foods and dishes. Therefore, the aim of this study was to evaluate the three-year school-based multicomponent intervention “Nutri-školica” in terms of changes in fruit and vegetable intake among primary school children. In addition, we estimated fruit and vegetable intake and observed whether socio-demographic characteristics, weight status, and lifestyle habits of the study population influenced fruit and vegetable intake at baseline.

## 2. Materials and Methods

### 2.1. Study Design and Settings

This was a prospective three-year longitudinal intervention study (from the 2018/2019 school year to 2020/2021) conducted in a sample of primary school students from 14 public primary schools in the city of Zagreb, Croatia.

The study takes place within the framework of the “Pilot Project: School Meals and Fruit and Vegetable Intake in Schools With and Without a Garden”, which is part of the Horizon 2020 project “Strengthening European Food Chain Sustain-ability by Quality and Procurement” (Strength2Food, H2020-SFS-2015-2, contract number 678024). Participants were recruited in three stages. In the first stage, schools were selected. A questionnaire was sent to the principals of all state schools (n = 107) in the city of Zagreb, asking them if they had a school garden and if they would be willing to participate in the study. Of all the schools with gardens (n = 35), 7 schools (20%) expressed interest in participating in the project. Then, the schools without gardens were selected using a statistical randomization algorithm. The selection criteria were: the two schools must not be in the same district, the school does not have a garden, and the schools were willing to participate. The algorithm was processed in C# programming language and Oracle Express database with PL/SQL. The minimum number of schools was set to 7 to achieve the best statistical power (ANOVA ω^2^ ≥ 0.14). The second stage was the selection of participants. Parents of all children (n = 1039) attending first grade in the 14 selected schools were informed of the aim and protocols of the study, and parents of 681 children (response rate = 66%) gave written informed consent to participate. There was no exclusion criterion for children to participate in the intervention in the selected schools. Finally, children in each school were assigned to an intervention or control group at class level. The principals of each school decided which class would participate in the intervention group and which in the control group. This was done in such a way that if there were an even number of classes, they were divided equally, and if there were an odd number of classes, one more class was assigned to the control group. Thus, the intervention group consisted of 19 classes with a total of 381 children, while the control group consisted of 21 classes with a total of 300 children. The intervention was conducted at the class level, and only children and parents from the intervention group had access to the materials during the intervention. Only one teacher from each class that participated in the intervention had access to the educational materials and did not distribute materials to other teachers and children who did not participate in the intervention.

This study was designed and conducted as a cross-sectional observational study in accordance with the Declaration of Helsinki and approved by the Ethics Committee of the School of Medicine, University of Zagreb (380-59-10106-19-11/307). Approvals for the implementation of the pilot project in primary schools were obtained from the relevant institutions (the Ethics Committee of the Institute for Medical Research and Occupational Health: 100-21/16-8; the Croatian Ministry of Science and Education and the Education and Teacher Training Agency: 602-01/16-01/00388).

### 2.2. Participants

Of the total 681 children, 28 children did not complete the intervention because they transferred to another school that was not included in the study. In addition, 431 (63.3%) completed the food intake frequency questionnaire before the intervention and 265 (38.9%) after the intervention. Analyses were performed based on the per-protocol approach. Therefore, the present study was conducted on a total of 259 children (38.0%) who completed the questionnaire at both time points. The sample size calculation was based on the impact of nutritional intervention on the change in fruit and vegetable intake, for the two intervention arms. It was calculated that 128 children would provide an appropriate sample size in order to obtain an acceptable 80% power at the α = 0.05 significance for the result interpretation (version 3.1.9.2; Heinrich Heine University Dusseldorf, Dusseldorf, Germany).

### 2.3. Intervention

The multicomponent school-based intervention titled “Nutri-školica” (Little nutrition school) was designed with the aim of increasing fruit and vegetable preferences and consumption among primary school students. The intervention was designed according to the 7 steps of programming [26] and was implemented in selected classes in 14 primary schools for three school years (2018/2019 to 2020/2021).

**Figure 1:**
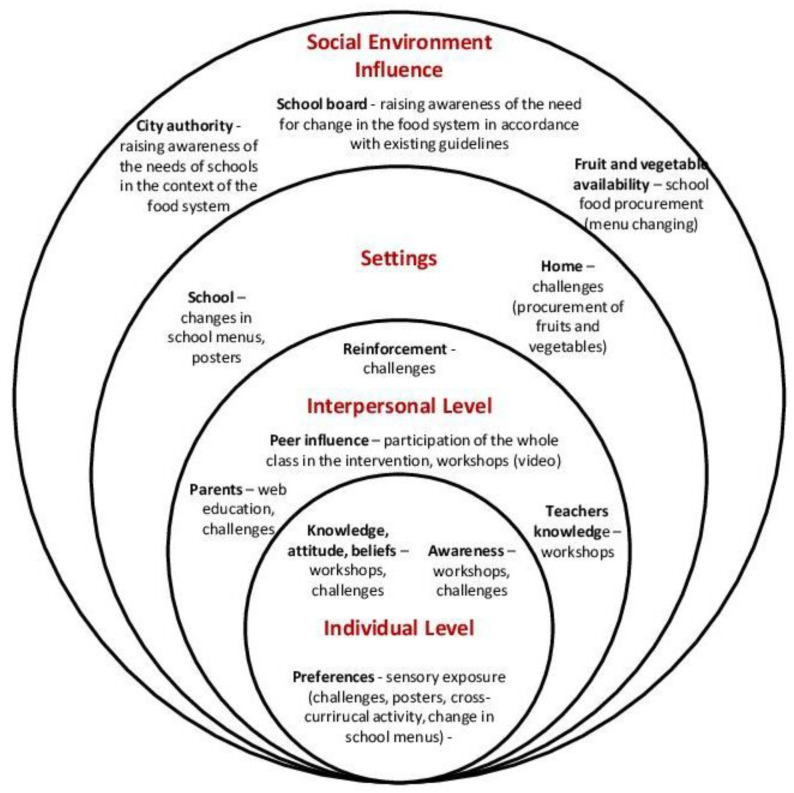
The socio-ecological framework for the intervention.

The intervention included activities at all levels of the socio-ecological model (Figure 1) [16,27]. The knowledge–attitude–behavior model, social cognitivist theory, and self-determination theory were selected to provide the theoretical framework of the eating behavior change intervention. The intervention consisted of 23 interactive classroom workshops lasting 45 min, 10 cross-curricular activities, 13 homework challenges, visual engagement with educational posters in classrooms, parent education via the website, and modification of existing and introduction of new dishes into the school food system. Interactive classroom workshops were conducted by a nutritionist with additional educational training. The workshops focused on healthy eating postulates, raising awareness of fruit and vegetable consumption, linking fruit and vegetable consumption to health benefits, eating seasonal and local fruits and vegetables, etc. The cross-curricular activities were carried out by primary school teachers and were designed to stimulate students to learn about fruit and vegetables through other school subjects (e.g., math problems with fruit and vegetables to count, a game for physical education where different balls are fruit, vegetables that must be arranged in individual containers…). The children were given 13 challenges to complete at home with their parents. The challenges were focused on taste exposure to encourage acceptance of fruit and vegetables [28,29,30], as it was not possible to conduct activities with sensory exposure in the classroom. For each completed challenge, the children received a sticker, and the children who collected all the stickers at the end of the school year received an additional prize. The educational posters used to decorate the classrooms for the purpose of visual exposure were created by nutritionists as educational materials or were made by the children as part of classroom activities. The official website was developed for parent education and includes blog posts focused on appropriate child nutrition, fruit and vegetable consumption, and advice on implementing healthy eating principles in the family. All educational materials (brochures, presentations, posters, teaching aids, etc.) were designed by the research team. Fidelity measures were obtained from research (number of educational activities conducted, number of children who participated in educational activities, number of children who completed challenges, number of educational posters in the classroom, number of emails addressed to parents with educational blog-posts) and from teachers (number of interdisciplinary activities conducted). In 19 intervention classes, 19 (83%) to 23 (100%) of educational workshops were conducted due to COVID-19 pandemic. Looking at the number of workshops a student attended, at least 15 (65%) to all 23 (100%) workshops were attended. This is because the workshops were held during regular classes and it was not possible to influence whether children were in school. However, the workshops were attended by teachers who, when the children returned to school, made up the missed workshops with them. The teacher conducted up to 4 (40%) cross-curricular activities. All educational posters, 5 in the second year and 3 in the third year of the intervention, were hung in classes from the time they were created/used in the workshops until the end of the school year in all 19 interventional classes. On average, children achieved about 84% of the challenges (46–100%), and there was not a single child who did not achieve a single challenge. On average per class, 22% of the children won all 13 challenges, 53% of the children with 10 to 12 challenges and 25% of the children with 6 to 9 challenges. All parents received 5 mails with 5 blog posts education. The number of parents who read the educational blog-posts was not recorded.

The primary outcome measures were change in fruit and vegetable intake and preferences, measured at baseline and at the end of the intervention. As a secondary outcome, the relationship between the characteristics of the children (dietary assessment, lifestyle assessment, socio-demographic characteristics and anthropometric characteristics) and the consumption and preference for fruits and vegetables estimated at baseline. The present study shows the primary and secondary outcomes related to fruit and vegetable intake.

### 2.4. Assessment of Fruit and Vegetable Intake

Fruit and vegetable intake was assessed using the semi-quantitative food frequency questionnaire to estimate fruit and vegetable intake among school-aged children [31]. The questionnaire consists of 18 questions reflecting fruit and vegetable intake in the last month. The results of the questionnaire indicate the daily intake in grams of 8 food groups (“fruit and fruit juices”, “fruit”, “vegetables, vegetable juices and dry legumes”, “vegetables and vegetable juices”, “vegetables”, “fruit, fruit juices, vegetables, vegetable juices and dry legumes”, “fruit, fruit juices, vegetables and vegetable juices” and “fruit and vegetables”). The definition of fruit and vegetables has been adopted by the World Health Organization [32]. The term vegetable refers to the edible parts of plants that are considered vegetables, fresh green legumes, mushrooms, seaweeds, and sprouts, while the term legume refers to the consumption of dry legumes (e.g., beans, lentils, chickpeas, etc.). The term fruit refers to fruit unless they are classified as vegetables (tomatoes, peppers, cucumbers, etc.), while 100% fruit juices were assessed with a question separate from fruit consumption. The total daily intake of fruit and vegetables was compared with the daily amount of 400 g recommended by the World Health Organization [1].

The questionnaire was distributed online to the parents of children enrolled in the study. They were advised to complete the questionnaire together with their children to obtain better insight into the frequency of the fruit and vegetable intake while the children were not under their supervision.

### 2.5. Anthropometric Data

The children’s height and weight were measured according to standard protocols in light athletic clothing and without shoes using a combined medical digital scale and sta-diometer (Seca, Type 877–217, Vogel & Halke Gmbh & Co., Hamburg, Germany) at baseline. Height was measured to the nearest 0.1 cm and weight to the nearest 0.1 kg. Body mass index (kgm^−2^) was calculated as body weight in kilograms divided by height in meters squared. Sex- and age-standardized z-scores for weight, height and body mass index were calculated for each child using AnthroPlus software [33]. Accordingly, the sex- and age-standardized z-score cutoffs suggested by the World Health Organization were used to estimate the weight status of the children [34].

### 2.6. Physical Activity Level

The level of physical activity of each child was estimated using the Physical Activity Questionnaire for Older Children (PAQ-C), which was validated for the Croatian child population [35,36]. The questionnaire was provided online at baseline to parents, and they were instructed to complete it with the help of their children. The questionnaire consists of 9 questions reflecting the children’s physical activity during the last week. The final score is a 5-point scale, with 1–2 classifying children as insufficiently physically active, 3 as moderately physically active, and 4–5 as very physically active.

### 2.7. General Questionnaire

Parents of all children completed an online questionnaire at baseline about the children’s date of birth, sex (choose one of the “male or female” options), household income, and lifestyle habits. Lifestyle habits included questions about sleep duration and screen time. Accordingly, average daily sleep time in minutes was calculated from 4 questions about the time the child usually goes to bed and wakes up on school days and the time the child usually goes to bed and wakes up on weekends [37,38]. In addition, average daily screen time in minutes was calculated from 2 questions about the number of hours their child watches TV, plays video games, etc., on school days and weekends [5,38,39].

### 2.8. Data Analysis

Analyses were performed using IBM SPSS Statistics v. 23.0, released 2015 (IBM SPSS Statistics for Windows, Version 23.0. Armonk, NY, USA: IBM Corp.). The continuous variables are presented as mean ± standard deviation and the categorical as number or percentage. Shapiro Wilk test was used to test the distribution of variables. Accordingly, for normally distributed data, the independent Student’s t test was used, and for non-parametric data, the Mann–Whitney U test was used to compare the differences between the control and the intervention group at the baseline of the study. The Chi-square test was used to compare the frequency of boys and girls between the control and the intervention group. In addition, point-biserial correlation was performed to observe the relationship between sex and food groups consumption, Kendall’s τ_b_ correlation between household income and food group, while Pearson correlation for normally distributed continuous variables, and Spearman’s correlation for non-normally distributed continuous variables were performed to observe the relationship between food groups consumption and weight status as well as lifestyle habits. Two-way ANCOVA with repeated measures adjusted for participation in school garden education was used to analyze differences in food groups consumption between the control and the intervention group before and after intervention. The level of significance was set at *p* < 0.05 for all the analyses.

## 3. Results

**Figure 2:**
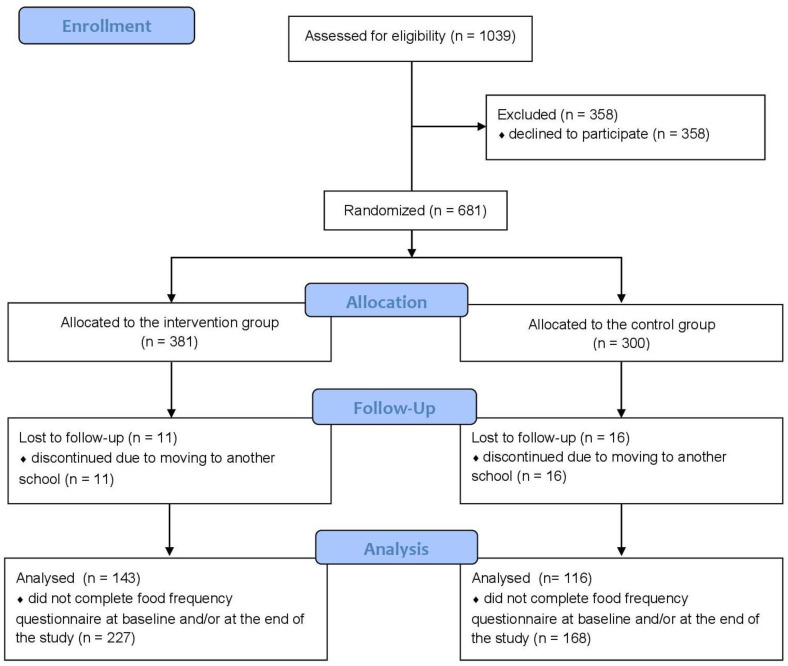
The CONSORT flow diagram for the resent study.

Of all allocated children (n = 681), a total of 654 completed the intervention (370 in the intervention group and 284 in the control group). Children (n = 27) dropped out of the study because they transferred to another school that was not included in the study (Figure 2). In addition, 227 children in the intervention group and 168 in the control group did not complete the food frequency questionnaire and were excluded from the data analysis. Therefore, data analysis was performed on a total of 259 children (143 in the intervention group and 116 in the control group) per protocol. There were no differences between the children who did not complete the questionnaire and those who did (Table S1).

Of the total study sample (n = 259), 49.8% were boys and 50.2% were girls. According to anthropometric measurements and estimated z-scores, the children had adequate weight, height, and body mass index (Table 1). The children were moderately active, spent approximately 2.5 h in front of a screen, and slept more than 9 h daily. About 55.2% of the children in the total sample were in the intervention group. There were no significant differences in sex, anthropometric measures, and lifestyle habits between the control and intervention groups, except for the z-score of body mass index for age. Namely, the children in the control group (0.2 ± 1.1) had a significantly lower (*p* = 0.034) mean z-score than the children in the intervention group (0.6 ± 1.2); however, both mean z-scores indicated that the children had adequate weight status. The majority of children were in the middle-income group by household income. In the overall sample, children’s fruit and vegetable intake at baseline was not related to sex, weight status, sleep duration, and screen time (Table 2). Higher levels of physical activity were associated with higher consumption of the vegetable food groups and the fruit and vegetable food groups but not with fruit consumption. Nevertheless, correlation coefficients indicate a weak positive correlation (< 0.300) between physical activity levels and consumption of food groups. In addition, it was found that children who lived in families with higher household income had higher fruit consumption, but again, the observed correlation was weak.

Fruit, fruit juices, vegetables, vegetable juices and dry legumes.

The results in Table 3 show that daily fruit consumption did not increase (*p* = 0.099), but daily fruit and fruit juice consumption increased significantly (*p* = 0.001) after the intervention in the intervention group (before: 198.6 ± 139.2 g; after: 280.3 ± 168.5 g) compared to the control group (before: 198.6 ± 139.2 g; after: 213.4 ± 136.0 g). Consumption of vegetables increased in the vegetable group (*p* = 0.010) and in the vegetable and vegetable juice group (*p* = 0.014), while no difference was observed between the control and intervention groups after the addition of dry legumes. Accordingly, the consumption of all three food groups including fruits and vegetables increased significantly in the intervention group compared to the control group after the intervention.

**Figure 3:**
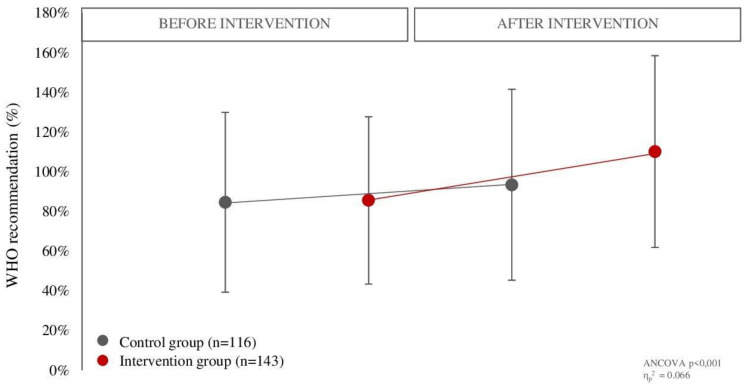
Comparison of the proportion in which children met the recommendations for the daily fruit and vegetable intake between the control and intervention groups before and after the intervention. Variable are presented as mean ± standard deviation. Difference between groups was tested using two-way ANCOVA with repeated measures adjusted for participation in school garden education (*p* < 0.05).

**Figure 4:**
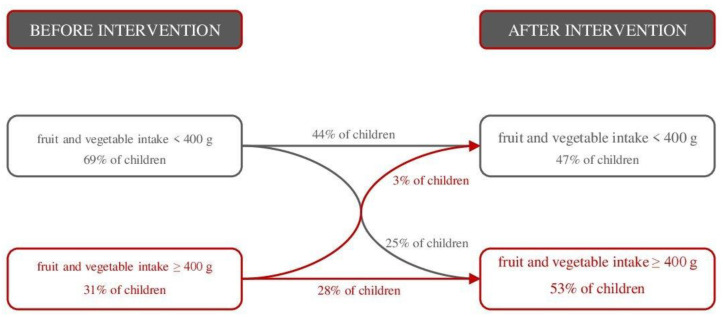
Distribution of children who met recommendation for daily fruit and vegetable intake in the intervention group (n = 143) before and after the intervention.

The changes in total daily fruit and vegetable intake were also reflected in the proportion of daily intake recommended by the World Health Organization (Figure 3). Thus, children in the intervention group (before: 83.0 ± 41.2% of the recommendation; after: 107.5 ± 46.7% of the recommendation) met the recommendation to a significantly greater extent (*p* < 0.001) than children in the control group (before: 84.6 ± 45.4% of the recommendation; after: 93.4 ± 48.1% of the recommendation) after the intervention. In addition, 25% of the children increased their fruit and vegetable intake to more than 400 g per day (Figure 4).

## 4. Discussion

To the best of our knowledge, this is the first study to evaluate the impact of a multicomponent nutrition intervention aimed at increasing the acceptance and consumption of fruit and vegetables among children in primary schools in Croatia. The present study indicates the success of the implemented intervention “Nutri -školica” and thus its potential positive use in the school system.

At baseline, mean daily fruit and vegetable intake in the total study population was 354.1 ± 180.9 g (4.4 ± 2.3 portions), of which approximately 221.7 ± 142.3 g (2.8 ± 1.8 portions) was fruit and 132.2 ± 72.7 g (1.7 ± 0.9 portions) was vegetables. Fruit and vegetable intake did not differ between the control and intervention groups at baseline. According to the World Health Organization, it is recommended to consume at least 400 g of fruit and vegetables per day [1]. In the present study, only 34% of the children in the total sample met the recommendation for fruit and vegetable intake. Although there are different definitions of fruit and vegetables, in the present study, fruit and vegetable intake is assessed according to the World Health Organization definition. Therefore, the term vegetable includes all fresh, frozen, cooked, and canned vegetables and 100% vegetable juice, excluding tuber vegetables and starchy roots (potatoes, sweet potatoes, yams, etc.) and dry legumes, and the term fruit includes all fresh, cooked, canned, and dried fruit and 100% fruit juice, excluding nuts [32]. Notwithstanding the differences in definitions of fruit and vegetables worldwide, and thus in methods of assessing consumption, it has been estimated that children consume relatively low fruits and vegetables and that only a minority of them consume fruit and vegetables daily [3,40]. In European countries, children aged 4 to 12 years consume between 229 and 404 g of fruit and vegetables per day, with the lowest intake recorded in Ireland and the highest in Denmark [41,42,43,44,45,46]. The average daily fruit and vegetable intake of the population in the present study is most similar to that of children in Italy (350 g) and Spain (341 g), which, like Croatia, belong to the Mediterranean region [42,46]. The studies conducted in America are difficult to compare with those conducted in Europe, since the consumption of fruit and vegetables is expressed in cups (250 mL) [47]. However, the data show that the majority of primary school children consume fruit (approximately 75.3% of children) and vegetables (approximately 90% of children) on the day they are offered [7].

According to the available literature, there are several biological and socioeconomic factors that may influence the fruit and vegetable intake in children. In the present study, we observed the relationship between six potential determinants to be used as covariates in further data analyses [32,48,49,50,51,52,53,54]. In the present study, there was no significant relationship between sex, z-score for body mass index, sleep duration, and screen time with fruit and vegetable intake. Only physical activity level was significantly correlated with fruit and vegetable intake, and household income was significantly correlated with fruit intake only, but both correlations were weak. The results of the present study are consistent with those of the available literature on sex and weight status [48,53,55]. In contrast, results from the available literature suggest that higher fruit and vegetable intake is associated with higher physical activity, longer sleep duration, shorter screen time, and higher socioeconomic status [54,56,57,58,59]. Despite the findings in the aforementioned studies, none of the observed determinants were included in further analysis in the present studies because there was no or only a very weak association with fruit and vegetable intake in our study population. The data analysis was adjusted for the additional education in the school garden, as it persisted in both the intervention and the control group due to the school recruitment protocol for the implementation of other activities within the Strength2Food project. Namely, education in the school garden can encourage fruit and vegetable intake in children [60,61].

The results of the present study suggest that the intervention can successfully increase fruit and vegetable intake in children. An increase in fruit and vegetable consumption was achieved by 89% of children in the intervention group, and 25% more children met the World Health Organization recommendations for daily fruit and vegetable consumption of 400 g [1]. The children in the intervention group consumed 80 g more fruit and fruit juices and 27 g more vegetables and vegetable juices daily, resulting in a 102 g increase in total daily fruit and vegetable consumption. Specifically, children in both groups similarly increased their intake of fruit over time, but children in the intervention group consumed significantly more fruit and fruit juices than children in the control group. This means that they increased their juices consumption significantly more than the children in the control group. Fruit juices refer to 100% fruit juice, not sugar-sweetened beverages. The average fruit juice consumption of the children increased for 30 mL; thus, children in the intervention group consumed 60 mL of juice per day after the intervention. This increase in fruit juice consumption does not present a risk for the development of chronic non-communicable diseases because daily consumption is still within the recommended amount. According to the recommendations of the World Health Organization, children aged 7–18 years can consume up to 100 mL of fruit juice for one serving of fruit within the five-portions-per-day recommendation [62], and according to the recommendations of the American Academy of Pediatrics, children can consume 100–130 mL of fruit juices per day [63]. Regarding the consumption of vegetable food groups, children in the intervention group consumed significantly more vegetables and vegetable juices compared with the control group. However, after the addition of dried legumes, consumption increased similarly in both groups. According to the available reviews and meta-analyses, about 65–75% of interventions had some success in increasing fruit and vegetable intake. In general, school-based interventions are thought to moderately increase fruit intake but weakly increase vegetable intake [9,11,12,15,64]. One of the review studies highlighted that the interventions increased fruit and vegetable intake by 0.30 to 0.99 servings in children [12]. In a later review study, a similar effect of the interventions was observed, with the interventions increasing fruit intake by about 80 g (0.5 cup or 1 medium fruit) and vegetable intake by 20–30 g (1/4 to 1/3 cup of vegetables) [13]. Considering that children should consume at least 400 g of fruit and vegetables per day [1], which corresponds to an average of 5 servings of 80 g, the present intervention, “Nutri-školica”, had a similar reach as the other interventions.

The findings from the present study add to the existing literature on how different intervention designs and implementations can affect fruit and vegetable intake among primary school children. In developing the intervention, we were guided by the fact that interventions with multiple components are more successful than interventions with only one component [11,13]. We also considered the results of previous studies suggesting that fruit and vegetable consumption can be increased through education of children and parents, peer and teacher influence, reinforcement, video-peer modeling, sensory exposure, and increasing the availability of fruits and vegetables through school menus [10,11,30,65,66,67,68,69]. It was expected that the increase in fruit and vegetable intake would be greater compared to previous studies; nevertheless, the results of the present intervention are not significantly different from previous interventions, which had a moderate effect on increasing fruit consumption and a weak effect on increasing vegetable consumption [13]. Given that this was a multi-component intervention, it was difficult to distinguish which of the components had an effect on changing fruit and vegetable intake. A possible lower impact of the intervention could be due to the COVID-19 pandemic, where the adequacy of the implementation of the whole intervention was reduced. Namely, we attempted to increase the availability of fruit and vegetables through the school menus, but this activity was not carried out to the end due to the COVID-19 pandemic. In addition, about 74% to 91% of education and up to 40% of cross-curricular activities were successfully conducted in schools. The COVID-19 pandemic could also have affected children’s eating habits. During the implementation of the intervention, the children were often isolated together with their parents at home. The impact of the COVID-19 pandemic was most pronounced in the fact that parents and children had more home-prepared cooked meals; however, the daily number of snacks increased in children. Accordingly, higher consumption of food with lower nutritional density prevailed over consumption of food with high nutritional density [70,71]. Furthermore, as children did not go to school and ate school meals, the positive impact of school meals on the daily fruit and vegetable intake could have been reduced, which can be more reflected in low-income families [72,73].

The intervention “Nutri-školica” is the first implemented school-based intervention in primary school in the city of Zagreb, which was evaluated in terms of the set goals. In the present study, we have presented the impact of the intervention on the change in fruit and vegetable intake, but further analysis of the change in preferences and their relationship is warranted. The present study has several strengths and limitations that should be acknowledged. One of the limitations is the way the respondents were divided into the control and intervention groups, as this was decided by principal. The study was analyzed using the per-protocol approach because a large proportion of children in both the control and intervention groups did not complete the food frequency questionnaire. Although there were no differences in socio-demographic characteristics, anthropometric measurements, and lifestyle habits between completers and non-completers, there is a possibility that children who completed both food frequency questionnaires were more favorable to fruit and vegetables which could be a potential source of bias. However, the sample size in the present study was adequate and can ensure sufficient statistical power. The children in the present study came from 14 schools in the City of Zagreb, which differed in terms of location (center and suburbs) and socioeconomic area according to the poverty area index. Data were analyzed shortly after the end of the intervention, and long-term follow-up is lacking. The data analysis was carried out with regard to the difference in the fruit and vegetable intake before and after the intervention in the intervention group compared to the control group. This type of analysis reduces the possible error caused by the increase in fruit and vegetable intake as children grow older. The “Nutri-školica” intervention was a multicomponent intervention designed in accordance with children’s need and the socio-cultural environment in which it was implemented, but because of the intervention design, it was difficult to determine which component had an impact. In future studies, it is necessary to design a study protocol and accompanying questionnaires that can determine which components influence changes in eating behavior and to what extent. Furthermore, due to the COVID-19 pandemic, not all activities could be fully implemented, especially the change in school menus. Changes in school menus should result in schools offering children an equal and appropriate amount of fruit and vegetables. Despite the varying amounts of fruit and vegetables offered to children by the schools, all schools had the same conditions for food procurement and participation in the School Scheme program. In Croatia, participating schools in the “School Scheme” program receive free-of-charge 100–150 g of fresh vegetables (tomatoes, carrots, radish and kohlrabi) or fruit (apples, pears, apricot, nectarine, peaches, plums, cherries, figs, mandarins, grapes, strawberry, raspberry and blackberries) per student once per week throughout the school year [74]. To assess fruit and vegetable intake, we used a questionnaire on frequency of consumption of fresh/cooked fruit and vegetables and composite dishes that reflected the frequency of fruit- and vegetable-containing foods and beverages consumed in the past month to minimize daily variation in children’s diets [32,75,76]. To minimize this potential error, parents along with their children completed the online food frequency questionnaire in late spring and summer when the availability of fruits and vegetables is similar [77]. To minimize the influence of children’s age and parents’ inability to assess children’s food consumption outside their supervision, parents completed the online food frequency questionnaire with their children. In addition, food frequency questionnaires were completed in late spring and summer when fruit and vegetable availability is similar. Finely, in Croatian nutrition guidelines for primary school children, it is recommended that fruit and vegetables should be consumed daily [22]; therefore, in the present study, we used recommendations of the World Health Organization for the evaluation of fruit and vegetable intake in children.

## 5. Conclusions

The present study showed that the intervention “Nutri-školica” had a moderate effect on fruit intake and a small effect on vegetable intake in primary school children. Children increased daily fruit and vegetable intake by approximately 102 g. About 89% of children who participated in the intervention increased fruit and vegetable intake, and 25% exceeded the World Health Organization’s recommended daily intake of 400 g. The impact of the present intervention is comparable to that of other global interventions.

## Figures and Tables

**Table 1:** Baseline characteristics of the study sample ^1^.

Parameter	Total Sample	Control	Intervention	*p* Value ^2^
Participants (n)	259	116	143	
Age (yr.)	7.7 ± 0.4	7.7 ± 0.3	7.7 ± 0.4	0.476
Sex (%):				
Boys	49.8	49.1	50.3	0.846
Girls	50.2	50.9	49.7	
Body height (cm)	135.5 ± 5.9	135.4 ± 6.2	135.5 ± 5.7	0.818
Body height for age z-score	0.9 ± 1.0	0.9 ± 1.1	0.9 ± 0.9	0.989
Body weight (kg)	31.6 ± 6.6	30.7 ± 5.9	32.2 ± 7.0	0.129
Body weight for age z-score	0.8 ± 1.1	0.7 ± 1.1	0.9 ± 1.1	0.089
Body mass index (kgm^−2^)	17.7 ± 2.7	16.6 ± 2.3	17.4 ± 2.9	0.060
Body mass index for age z-score	0.4 ± 1.2	0.2 ± 1.1	0.6 ± 1.2	0.034
Physical activity level	3.1 ± 0.6	3.1 ± 0.7	3.1 ± 0.6	0.754
Sleep time (min/day)	585.2 ± 33.3	580.9 ± 34.2	589.1 ± 32.2	0.098
Screen time (min/day)	137.9 ± 69.9	141.8 ± 76.8	134.3 ± 63.2	0.992
Household income (%):				
<5000.00 kn (664.92 €)	2.2	3.4	1.0	0.750
5000.00–8000.00 kn (664.93–1063.87 €)	6.6	5.7	7.3	
8000.01–14,000.00 kn (1063.88–1861.77 €)	35.0	34.5	35.4	
14,000.01–18,000.00 kn (1861.78–2393.70 €)	21.9	19.5	24.0	
≥18,000.01 kn (≥2393.71 €)	34.4	36.8	32.3	

^1^ Continuous variable are presented as mean ± standard deviation, while categorical as number or percentages. ^2^ Differences between groups were tested using Student’s *t* test for continuous parametric variables, Mann–Whitney U test for continuous non-parametric variables, and Chi-square test for categorical variables (*p* < 0.05).

**Table 2:** Correlation between children’s characteristics and fruit and vegetable food groups intake at baseline (n = 259).

Food Groups	Sex ^1^	Body Mass Index for Age z-Score ^2^	Physical Activity Level ^2^	Sleep Time ^2^	Screen Time ^2^	Household Income ^3^
Fruit:						
Fruit	0.096	0.054	0.114	0.048	−0.014	0.125 *
Fruit and fruit juices	0.077	0.029	0.126	0.042	0.027	0.097
Vegetables:						
Vegetables	0.043	0.005	0.166 *	0.092	0.068	0.056
Vegetables and vegetable juices	0.034	0.013	0.177 *	0.099	0.068	0.057
Vegetables, vegetable juices and dry legumes	0.035	0.011	0.167 *	0.098	0.066	0.061
Fruit and vegetables:						
Fruit and vegetables	0.093	0.045	0.138 *	0.077	0.040	0.103
Fruit, fruit juices, vegetables and vegetable juices	0.073	0.028	0.142 *	0.072	0.062	0.107
Fruit, fruit juices, vegetables, vegetable juices and dry legumes	0.059	0.026	0.139 *	0.072	0.064	0.108

^1^ Point-biserial correlation. ^2^ Pearson correlation for parametric variables and Spearman’s correlation for non-parametric variables. ^3^ Kendall’s τ_b_ correlation. * *p* < 0.05.

**Table 3:** Comparison of children’s fruit and vegetable intake between the control group and the intervention group before and after the intervention ^1^.

Food Groups	Before Intervention		After Intervention		*p* Value ^2^	η_p_^2^
	Control Group (n = 116)	Intervention Group (n = 143)	Control Group (n = 116)	Intervention Group (n = 143)		
Fruit:						
Fruit	188.6 ± 141.4 (162.9–219.9)	171.7 ± 123.5 (149.7–193.2)	211.2 ± 144.4 (181.5–238.4)	216.2 ± 143.1 (193.3–240.9)	0.099	0.011
Fruit and fruit juices	198.6 ± 139.2 (173.8–224.4)	198.6 ± 139.2 (186.2–231.7)	213.4 ± 136.0 (184.6–241.5)	280.3 ± 168.5 (255.0–306.2)	0.001	0.040
Vegetables:						
Vegetables	134.5 ± 82.2 (121.7–146.5)	119.9 ± 53.3 109.0–113.4)	142.6 ± 84.7 (128.9–156.9)	147.0 ± 68.9 (134.1–159.3)	0.010	0.026
Vegetables and vegetable juices	137.1 ± 85.4 (123.5–149.7)	122.7 ± 57.7 (111.3–134.9)	145.2 ± 87.4 (131.1–159.9)	149.8 ± 70.5 (136.6–162.5)	0.014	0.023
Vegetables, vegetable juices and dry legumes	174.9 ± 135.8 (154.6–191.2)	127.3 ± 60.5 (112.5–145.4)	206.8 ± 151.5 (186.3–228.5)	155.6 ± 72.9 (136.1–174.1)	0.580	0.001
Fruit and vegetables:						
Fruit and vegetables	320.3 ± 177.8 (289.9–349.8)	291.5 ± 149.4 (264.9–318.8)	354.6 ± 187.5 (321.9–386.4)	362.8 ± 165.4 (334.2–392.3)	0.011	0.023
Fruit, fruit juices, vegetables and vegetable juices	350.2 ± 187.5 (317.4–381.7)	332.1 ± 164.9 (303.6–361.6)	382.6 ± 196.8 (347.1–417.3)	430.1 ± 186.7 (398.8–642.1)	<0.001	0.078
Fruit, fruit juices, vegetables, vegetable juices and dry legumes	313.5 ± 188.4 (282.4–347.2)	336.7 ± 166.7 (306.5–364.7)	321.8 ± 224.4 (283.9–359.1)	435.9 ± 187.2 (402.3–470.0)	0.002	0.074

^1^ Variable are presented as mean ± standard deviation (95% CI). ^2^ Differences between groups were tested using two-way ANCOVA with repeated measures adjusted for participation in school garden education (*p* < 0.05).

## Data Availability

The data are available upon request from A.I.

## Supplementary Materials

The following supporting information can be downloaded at: https://www.mdpi.com/article/10.3390/nu14194197/s1, Table S1: Comparison of characteristics of non-completers and completers in the control and intervention groups at baseline.
