# Supplementary material for: Increasing Fruit and Vegetable Intake of Primary School Children in a Quasi-Randomized Trial: Evaluation of the Three-Year School-Based Multicomponent Intervention
Source: Nutrients. 2022 Oct 8;14(19):4197. doi: 10.3390/nu14194197 (PMC9571246; doi:10.3390/nu14194197)
Supplement: Supplementary file 1 [file nutrients-14-04197-s001.zip › nutrients-1935261-supplementary.pdf]

**Table S1.** Comparison characteristics of non-completers and completers in the control and intervention groups at baseline [1].

| Parameter | Control group (n=300) | | | Intervention group (n=381) | | |
|---|---|---|---|---|---|---|
| | Non-completers | Completers | p Value [2] | Non-completers | Completers | p Value [2] |
| **Participants (n)** | 184 | 116 | -[3] | 238 | 143 | -[3] |
| **Age (yr.)** | 7.7 ± 0.3 | 7.7 ± 0.3 | 0.795 | 8.6 ± 0.3 | 7.7 ± 0.4 | 0,251 |
| **Sex (%):** | | | | | | |
| Boys | 51.6 | 49.1 | 0.812 | 48.9 | 50.3 | 0.791 |
| Girls | 48.4 | 50.9 | | 51.1 | 49.7 | |
| **Body height (cm)** | 134.8 ± 6.0 | 135.4 ± 6.2 | 0.322 | 135.5 ± 5.7 | 135.5 ± 5.7 | 0,949 |
| **Body height-for-age z-score** | 0.8 ± 1.0 | 0.9 ± 1.1 | 0.157 | 0.9 ± 0.9 | 0.9 ± 0.9 | 0,776 |
| **Body weight (kg)** | 31.2 ± 7.2 | 30.7 ± 5.9 | 0.550 | 31.7 ± 6.6 | 32.2 ± 7.0 | 0.432 |
| **Body weight-for-age z-score** | 0.7 ± 1.1 | 0.7 ± 1.1 | 0.918 | 0.9 ± 1.1 | 0.9 ± 1.1 | 0.481 |
| **Body mass index (kgm$^{-2}$)** | 17.0 ± 2.8 | 16.6 ± 2.3 | 0.159 | 17.1 ± 2.8 | 17.4 ± 2.9 | 0.373 |
| **Body mass index-for-age z-score** | 0.4 ± 1.2 | 0.2 ± 1.1 | 0.217 | 0.4 ± 1.2 | 0.6 ± 1.2 | 0.323 |
| **Physical activity level** | 3.0 ± 0.5 | 3.1 ± 0.7 | 0.986 | 3.0 ± 0.5 | 3.1 ± 0.6 | 0,923 |
| **Sleep time (min/day)** | 585.4 ± 23.9 | 580.9 ± 34.2 | 0.599 | 590.3 ± 33.5 | 589.1 ± 32.2 | 0,897 |
| **Screen time (min/day)** | 120.5 ± 50.8 | 141.8 ± 76.8 | 0.325 | 146.8 ± 82.1 | 134.3 ± 63.2 | 0.913 |
| **Household income (%):** | | | | | | |
| < 5000.00 kn (664.92 €) | 0.0 | 3.4 | | 8.0 | 1.0 | |
| 5000,00 kn – 8000,00 kn (664.93 – 1063.87 €) | 17.2 | 5.7 | | 8.0 | 7.3 | |
| 8000,01 kn – 14000,00 kn (1063.88 – 1861.77 €) | 31.0 | 34.5 | 0,351 | 32.0 | 35.4 | 0.420 |
| 14000,01 kn – 18000,00 kn (1861.78 – 2393.70 €) | 24.2 | 19.5 | | 24.0 | 24.0 | |
| ≥ 18000,01 kn (≥ 2393.71 €) | 27.6 | 36.8 | | 28.0 | 32.3 | |

[1] Continuous variable are presented as mean ± standard deviation, while categorical as number or percentages. [2] Differences between groups were tested using Student's t-test for continuous parametric variables, Mann-Whitney U test for continuous non-parametric variables and Chi-square test for categorical variables ($p < 0.05$). [3] Statistical analyzes was not performed.
